# Bortezomib initiates endoplasmic reticulum stress, elicits autophagy and death in *Echinococcus granulosus* larval stage

**DOI:** 10.1371/journal.pone.0181528

**Published:** 2017-08-17

**Authors:** María Celeste Nicolao, Julia A. Loos, Christian Rodriguez Rodrigues, Viviana Beas, Andrea C. Cumino

**Affiliations:** 1 Laboratorio de Zoonosis Parasitarias, Departamento de Biología, Facultad de Ciencias Exactas y Naturales, Universidad Nacional de Mar del Plata (UNMdP), Funes, Nivel Cero, Mar del Plata, Argentina; 2 Consejo Nacional de Investigaciones Científicas y Técnicas (CONICET), Argentina; 3 Departamento de Química, Facultad de Ciencias Exactas y Naturales, Universidad Nacional de Mar del Plata (UNMdP), Funes, Nivel 2, Mar del Plata, Argentina; 4 Hospital Privado de Comunidad, Mar del Plata, Buenos Aires, Argentina; Seconda Universita degli Studi di Napoli, ITALY

## Abstract

Cystic echinococcosis (CE) is a worldwide distributed helminthic zoonosis caused by *Echinococcus granulosus*. Benzimidazole derivatives are currently the only drugs for chemotherapeutic treatment of CE. However, their low efficacy and the adverse effects encourage the search for new therapeutic targets. We evaluated the *in vitro* efficacy of Bortezomib (Bz), a proteasome inhibitor, in the larval stage of the parasite. After 96 h, Bz showed potent deleterious effects at a concentration of 5 μM and 0.5 μM in protoscoleces and metacestodes, respectively (P < 0.05). After 48 h of exposure to this drug, it was triggered a mRNA overexpression of chaperones (Eg-*grp*78 and Eg-calnexin) and of Eg-*ire*2/Eg-*xbp*1 (the conserved UPR pathway branch) in protoscoleces. No changes were detected in the transcriptional expression of chaperones in Bz-treated metacestodes, thus allowing ER stress to be evident and viability to highly decrease in comparison with protoscoleces. We also found that Bz treatment activated the autophagic process in both larval forms. These facts were evidenced by the increase in the amount of transcripts of the autophagy related genes (Eg-*atg*6, Eg-*atg*8, Eg-*atg*12, Eg-*atg*16) together with the increase in Eg-Atg8-II detected by western blot and by *in toto* immunofluorescence labeling. It was further confirmed by direct observation of autophagic structures by electronic microscopy. Finally, in order to determine the impact of autophagy induction on *Echinococcus* cell viability, we evaluated the efficacy of Bz in combination with rapamycin and a synergistic cytotoxic effect on protoscolex viability was observed when both drugs were used together. In conclusion, our findings demonstrated that Bz induced endoplasmic reticulum stress, autophagy and subsequent death allowing to identify unstudied parasite-host pathways that could provide a new insight for control of parasitic diseases.

## 1. Introduction

*Echinococcus granulosus* (dog tapeworm) is one of the three major cestoda which affect humans. It can cause hydatidosis or cystic echinococcosis (CE), which is still a public health issue with worldwide distribution [1]. The larval stage resides in the viscera of human and mammalian intermediate hosts as fluid-filled cysts, with protoscoleces budding from the germinal cell layer. It is surrounded by a very prominent outer acellular and carbohydrate-rich laminated layer synthesized by the parasite at the expense of its secretory activity [2]. Since over 20% of CE cases failed to be cured when choosing chemotherapy based on benzimidazolic drugs as main and only treatment, it is a dire need to find novel therapeutic strategies centered on new target identification [3].

In the secretory pathway, the endoplasmic reticulum (ER) is the central organelle of eukaryotic cells which participates in calcium handling and storage and protein synthesis, folding and transportation [4]. Perturbations in ER functions generate ER stress, which triggers the unfolded protein response (UPR), an intracellular signal transduction pathway designed to restore protein homeostasis [5]. Experimentally, ER stress is commonly initiated by alterations in synthesis and maturation of proteins, or by stimulation of accumulation of misfolded proteins to overcome UPR. This latter condition is precisely a consequence of the action of bortezomib (Bz, PS-341), a synthetic boronic acid dipeptide which operates as a selective and effective inhibitor of the 20S catalytic core particle of the proteasome, a multiproteinase complex responsible of the degradation of ubiquitinated proteins [6–7]. Specifically, Bz binds and inhibits the catalytic site of the β5-subunit (PSMB5) with chymotrypsin-like activity and also the caspase-like activity harbored by the β1 (PSMB6) subunit. At higher concentrations, the drug inhibits trypsin-like proteolytic activity facilitated by β2 (PSMB7) proteasome subunits [8].

Therefore, disruption of proteasome activity causes accumulation of degradable proteins and constitutive ER stress blocking the cellular growth and division and leading to cell death by apoptosis and/or autophagy [9–11]. As Bz showed selective cytotoxicity to cancer cells compared with normal cells in both *in vitro* and *in vivo* assays [6], it has been approved as a chemotherapeutic and anti-inflammatory drug [12–13]. Thus, Bz is used for the treatment of patients with a wide range of hematologic malignancies [14], also demonstrating to have cytotoxic effects on breast, colorectal, ovarian, pancreatic, lung and oral cancer cells [15]. Interestingly, Bz was also tested for *in vitro* activity against different protozoan parasites such as *Plasmodium* and *Trypanosoma* [16–17]. Recently, Bz was assayed as a potential candidate against the metacestode life-stage of *Echinococcus multilocularis* (fox tapeworm), the causative cestode of alveolar echinococcosis, a zoonosis limited to the northern hemisphere [18]. Previously, Tsai et al. [19] had identified all proteasome subunits as a top predicted targets based on proteome data in this cestode. Simultaneously, Zheng et al. [20] had reported the gene expression of the 14 catalytic subunits of the proteasome, differentially expressed among oncospheres, protoscoleces, cyst membrane and adult worms of *E*. *granulosus*, verifying a high expression level of β5 subunit in the metacestode form.

On the other hand, other genes identified in the *Echinococcus* genome are those that code for the glucose-regulated proteins (Grps), which are indicators of the onset of ER stress [19–23]. Grps are transcriptional induced as part of the UPR-associated genes in different organisms [5, 24], but this issue remains unknown in cestodes. Upon ER stress, Grp78 (also known as Immunoglobulin Binding Protein,-BiP- or 70 kDa Heat Shock Protein 5 -HSPA5-) is titrated away through binding to misfolded proteins, causing dissociation from the lumenal domains and activation of the stress transducers as PKR-like ER Kinase (PERK), Activating transcription Factor 6 (ATF6) and Inositol-Requiring Enzyme 1 (IRE1) [21, 25]. During UPR activation, other calcium-binding chaperones such as calnexin (Cnx) and calreticulin (Crt) are overexpressed. These are lectin-like integral membrane or luminal proteins, respectively, which play an important role in the folding and assembly of secreted and cell membrane-associated glycoproteins [26].

In view of the differences in the parasitic developmental rate and pathogenicity between *E*. *granulosus y E*. *multilocularis*, we examined the impact of *in vitro* pharmacological treatment with Bz on *E*. *granulosus* larval stage viability, analyzing the possible induction of UPR in this cestode. Previously, we had determined that rapamycin is an effective anti-echinococcal agent and an autophagy inducer which allowed us to identify TORC1-controlled events in the parasite [27–28]. Given that, proteasomal and autophagic-lysosomal degradation are two parallel cytoplasmic catabolic pathways that may be critical to maintain proteostasis in the cell [29]. Another aim was to analyze the occurrence of autophagy and verify the potential therapeutic effects upon combining both drugs. In this way, a better comprehension of the mechanisms that coordinate the ER stress responses may help to plan potential strategies for therapeutic benefit against *Echinococcus* sp.

## 2. Materials and methods

### Ethics statement

The animal study was performed in strict accordance with National Health Service and Food Quality (SENASA) guidelines, Argentina and with the 2011 revised form of The Guide for the Care and Use of Laboratory Animals published by the U.S. National Institutes of Health. All the experimental protocols were reviewed and approved by the Animal Experimental Committee at the Faculty of Exact and Natural Sciences, Mar del Plata University (permit number: 2555-08-16). Unnecessary animal suffering was avoided throughout the study.

### Experimental animals

Twenty pathogen-free female CF-1 mice weighing 28–35 g were supplied by the SENASA. The animals were housed in standard polyethylene cages (five mice per cage) with sawdust (wooden flakes) as nesting material, under controlled laboratory conditions (temperature ±20°C, 12 hour light/12 hour dark with lights off at 8.00 p.m.). Mice were allowed to acclimatize for one week before starting the experiment. Water and food pellets were provided *ad libitum* during the study period. Every 3 days, animals were placed into a clean cage with fresh sawdust. The health and well-being of the mice was monitored daily. *E*. *granulosus* metacestodes were obtained from mice after the infection with 0.5 ml of protoscolex suspension, which was injected into the peritoneal cavity. For each experiment, five experimentally infected mice were killed at 6 months post infection.

### *In vitro* culture of protoscoleces and metacestodes

*Echinococcus granulosus* protoscoleces were removed aseptically from hydatid cysts of vicera of infected cattle presented for routine slaughter at the abattoir in the province of Buenos Aires, Argentina. Protoscolex *in vitro* culture (n = 3,000/9.5cm^2^), pharmacological treatment and viability assays were performed as previously described [27]. Otherwise, *E*. *granulosus* metacestodes (10–20 cysts for each drug treatment) were obtained from the peritoneal cavities of CF-1 mice after intraperitoneal infection with protoscoleces [30]. *In vitro* protoscolex and metacestode treatments were assayed with rapamycin (Rm, Calbiochem, USA) and Bortezomib (Bz, Janssen-Cilag, Germany) dissolved in dimethyl sulfoxide (DMSO). Bortezomib was assayed at 0.1, 0.5, 1, 2.5, 5 and 10 μM as final concentrations in metacestodes and at 0.5, 5, 10, 20, 30 and 50 μM in protoscoleces. *In vitro* incubations were performed at 37° C and the drugs were replenished every 3 days during the complete experiment. For *in vitro* experiments, parasites were recovered and used for immunohistochemistry studies or they were washed with sterile and RNAse-free PBS and they were conserved at −80°C until experimental use for molecular experiments. Each experiment was assayed for three replicates and repeated three times. For scanning and transmission electron microscopy (SEM and TEM), samples were taken every 24 h and processed [27–28]. Samples were fixed with 3% glutaraldehyde in sodium cacodylate buffer for 24 h at 4°C. For SEM analysis, the specimens were dehydrated and finally immersed in hexamethyl-disilazane for 5 min, 1 h and 24 h. They were then sputter coated with gold (100 Å thick) and inspected on a JEOL JSM-6460 LV scanning electron microscope at 15 kV. For TEM analysis, samples were post-fixed in 2% OsO4 in cacodylate buffer, dehydrated in a graded acetone series and subsequently embedded in resin epoxi and examined with a JEM 1200 EX II (JEOL Ltd., Tokio, Japón) transmission electron microscope at 80 kV.

### Bioinformatic analyses and structure prediction

For phylogenetic studies and inferences of homology, sequences were obtained from the non-redundant protein databases (http://www.ncbi.nlm.nih.gov; http://www.sanger.ac.uk, and http://www.nematodes.org/NeglectedGenomes/Lopho/Loph_blast.php). Orthologs were selected based on reciprocal best BLAST hits [31–32], using an E-value cut-off of 10^_25^. Multiple protein sequence alignments were performed using ClustalW (http://www.ebi.ac.uk/Tools/msa/clustalo). Modelling of tertiary structures was obtained from the deduced primary structure using the Gen-THREADER (SWISS-PROT). Analyses of hydrophobicity profile was returned by ProtScale (http://web.expasy.org/protscale/). Prediction of transmembrane regions and membrane topology (http://www.cbs.dtu.dk/services/TMHMM and http://www.enzim.hu/hmmtop/), nuclear localization signal (http://nls-mapper.iab.keio.ac.jp/cgi-bin/NLS_Mapper_form.cgi) and golgi and endoplasmic reticulum localization signal (https://rostlab.org/services/ERGolgiDB/index.html and http://ccb.imb.uq.edu.au/golgi/golgi_predictor.shtml) were performed.

### Expression analysis of autophagic genes, Eg-*grp*78, Eg-*calnexin*, Eg-*ire*2 and Eg-*xbp*1

Different RNA extractions were carried out as previously described [27] from control and treated protoscoleces and metacestodes incubated with drugs as documented above. For reverse transcriptase PCR (RT-PCR) and reverse transcriptase quantitative-PCR (RT-qPCR) analysis, total RNA treated with DNase (RQ1 RNase-free DNase; Promega) was reverse transcribed using a mixture of Moloney murine leukemia virus reverse transcriptase (Promega), M-MLV RT Buffer (Promega), 30 pM oligo dT, 2.5 mM dNTPs and specific primers for Eg-*atg*1 to Eg-*atg*9, Eg-*atg*12, Eg-*atg*16 and Eg-*atg*18 [28]. The primers designed for Eg-*grp*78/*bip*, Eg-*calnexin*, Eg-*ire*2 and Eg-*xbp*1 gene detection were Eg-*grp*78-f (5´- CATCGCAAATGACCAAGGCAATCGTATAAC-3´), Eg-*grp*78-r (5´- GATAATTCGCAACACAGTCAGACCAGCAATG-3´), Eg-*cnx*-f (5´- GGTGCATACATTAAACTGTTATCTGCCTC-3´), Eg-*cnx*-r (5´- GCATAGTGGCATCTTCATCTATAACGAATTG-3´), Eg-*ire*2-f (5´- GCGTCGCGTTGAGTTGGAGAAAAGTTG-3´), Eg-*ire*2-r (5´- CTCCAGGATAAAGATGTTGCTCGGCTTG-3´), Eg-*xbp*1-f (5´- GACTTTTTAACTGAAGAGGAGAAGGTAC-3´) and Eg-*xbp*1-r (5´- GACAAAAGAGAGGCAGATCAGAAGTAG-3´) whose amplicon sizes were 439, 475, 322 and 348 bp, respectively. Comparative steady-state mRNA levels were determined after normalization to the actin gene expression as described [33]. PCR conditions were as follows: a single step at 95°C for 5 min plus 30 cycles at 95°C for 30 s min, 50°C for 1 min, 72°C for 1 min plus a single step at 72°C for 10 min. Once the optimal amount of input RNA and an appropriate number of cycles were determined for each gene product, PCR amplification occurred in the linear range. The size of amplicons was identified using a 1 Kb DNA ladder (Promega) in agarose gel electrophoresis. In addition, RTq-PCR experiments were carried out as described in Cumino et al. [33]. The PCR program was at 95°C (10 min), 30 cycles at 95°C for 15 s, 50°C for 30 s, and 72°C for 30 s. Product identification was confirmed by a melting curve analysis and visualized on agarose gels. The relative rate of each cDNA was normalized using *act*I (see above). Data analyses for a relative quantification of gene expression were performed by the comparative Ct (threshold cycle) method.

### Western blot analysis and immunohistochemistry

Proteins were separated by SDS-PAGE on 15% polyacrylamide gels for Eg-Atg-8 analysis [28]. Polypeptides were visualized with Coomassie Blue or electroblotted onto nitrocellulose membranes (HyBond C; Amersham), which were probed with primary rabbit polyclonal antibody directed against the N-terminus of human LC3 (MAP LC3β antibody H-50, Santa Cruz sc-28266, USA, 1:1000 dilution) or with primary mouse monoclonal antibody of human actin (JLA-20, Developmental Studies Hybridoma Bank-DSHB, USA, 1:2000 dilution) as a control for protein loading. Then, the membranes were incubated with anti-rabbit and anti-mouse immunoglobulin G (IgG) conjugated with alkaline phosphatase (Bio-Rad 170–6518 and 170–6520, respectively, USA, 1:500 dilution), and finally reveled with 5-Bromo-4-chloro-3-indolyl Phosphate/Nitroblue Tetrazolium.

For *in toto* immunohistochemistry, protoscoleces were fixed in 4% (w/v) paraformaldehyde in 0.1 M Phosphate-Buffered Saline (PBS, pH 7.4) for 4 h at 4 °C, and then washed for 24 h at 4 °C in permeabilizing solution (PBS at pH 7.4 containing 0.3% v/v Triton X-100 and 0.5% w/v Bovine Serum Albumin–BSA-). Control and pharmacologically treated protoscoleces were incubated for 5 days at 4 °C with the same primary antibody (1:50 dilution), and washed with PBS for 24 h at 4 °C. Finally, protoscoleces were incubated with goat anti-rabbit IgG conjugated with Alexa 488 for 24 h at 4°C, washed and counterstained with 2 μg ml^-1^ propidium iodide (Molecular Probes P-3566, Argentina, to observe all cell nuclei under optimal contrast conditions). They were observed with an inverted confocal laser scanning microscope (Nikon, Confocal Microscope C1). Negative controls consisted of omission of primary antibody for both experiments. The intensities of green (excitation/emission wavelength = 485/538 nm) fluorescence were analyzed for 20× images from control and treated-protoscoleces. Optical sections were obtained at increments of 0.3 mm in the z-axis and were digitized with a scanning mode format. The fluorescence intensity at the selected areas, linearly correlated with the number of pixels, was quantitatively analyzed using standard imaging analysis from Image J software (NIH, http://rsb.info.nih.gov/ij/).

### Statistics

The mRNA expression in protoscoleces and metacestodes was analyzed with the Wilcoxon signed rank nonparametric test. Data within experiments were compared; significance was determined using the student’s t test and P < 0.05 was considered statistically significant. All data are shown as the arithmetic mean ± SEM.

## 3. Results

### *In vitro* activity of bortezomib against *E*. *granulosus* larval stages

**Fig 1:**
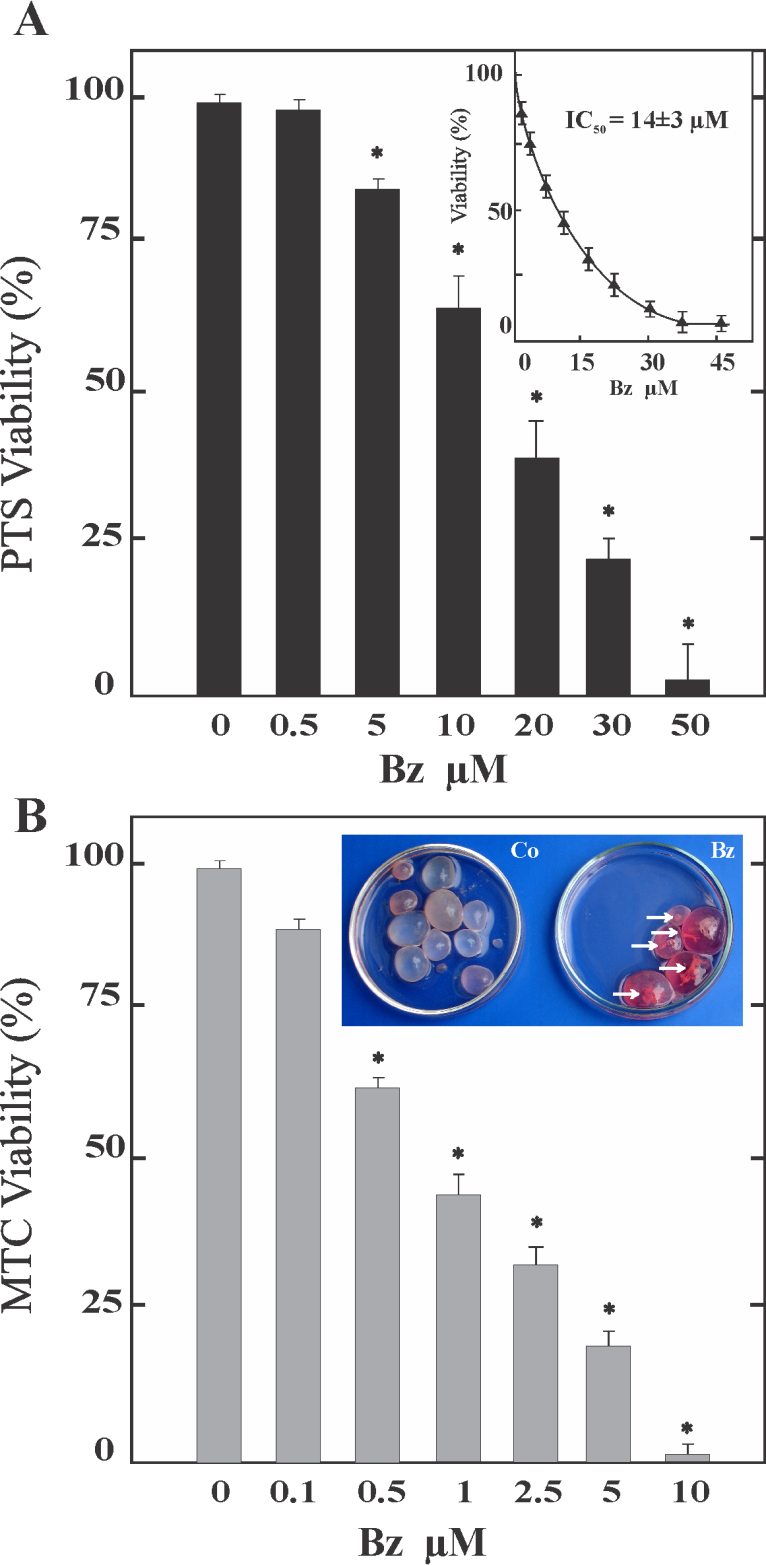
Effect of bortezomib (Bz) on viability of *E*. *granulosus* larval stages. Viability of protoscoleces (A) and metacestodes (B) incubated with different concentrations of Bz for 4 days. Data are the mean ± S.D. of three independent experiments. *Statistically significant difference (P < 0.05) compared with control. (A) The IC_50_ is shown in the inset. (B inset) Macroscopical damage of treated metacestodes with 0.1 μM of Bz during 6 days showing increased permeability (culture medium inside cysts) and collapsed germinal layer (arrows), (Co) control metacestodes without morphological changes.

Anti-echinococcal activity of Bz was tested in metacestodes and protoscoleces maintained *in vitro* and observed over 1 and 2 weeks, respectively. Viability decreased as a function of drug concentration in both larval stages (Fig 1). Bortezomib had effect at 5 μM and 0.5 μM after 96 h of treatment in protoscoleces and metacestodes, reducing the viability with 10 μM to 60 ± 5% and 100% (detachment of the germinal layer) after 96 h, respectively (Fig 1A and 1B). The protoscolex mortality rate was 100% after incubation during 8 days with 10 μM Bz and the calculated 50% inhibitory concentration (IC_50_) against protoscoleces was 14 ± 3 μM (Fig 1A, inset). Metacestodes incubated with 0.1 μM of Bz presented detachment of the germinal layer in 100% of cysts after 6 days (Fig 1B, inset). Untreated protoscoleces and metacestodes remained at least 99 ± 1.0% viable during the complete experiment.

### Occurrence of UPR-related genes in *E*. *granulosus*

To evaluate the occurrence of UPR transducers such as IRE-1, XBP-1, ATF6, PERK and ATF4 extensive tBLASTn searches were carried out in the assembled *E*. *granulosus* genome. We identified a single sequence for *atf*6, *ire* and *xbp*-1 genes which were annotated as EgrG_000116500 (CDS21671.1), EgrG_000182000 (CDS21410.1), EgrG_000843400 (CDS16021.1) in the GeneDB database, respectively. However, *perk* and *atf*4 homologs were not identified in the cestode genome. The predicted polypeptide sequence of Eg-IRE aligned with 27 and 26% identity with *Homo sapiens* (Q76MJ5.4) and *Drosophila melanogaster* (ABW08704.1) orthologs while the predicted Eg-XBP-1 aligned with 43 and 40% identity with *H*. *sapiens* (AAH12841.1) and *D*. *melanogaster* (AAF46681.2) orthologs, respectively. The predicted Eg-ATF6 sequence aligned with 44 and 49% identity with *H*. *sapiens* (NP_031374.2) and *D*. *melanogaster* (NP_995745.1) orthologs, respectively. The structure analysis of these proteins showed that all domains corresponding to specific functions were conserved (S1 Fig). Additionally, molecular chaperones that reside in the ER as Eg-Grp78 [23] presented a high degree of identity and conserved domains with *H*. *sapiens* (P11021.2, 76%) and *D*. *melanogaster* (P29844.2, 78%) orthologs. Also, the predicted polypeptide sequences of Eg-Calnexin and Eg-Calreticulin [34] aligned with 54 and 58% identity with *H*. *sapiens* orthologs, respectively (S1 Fig and data not shown).

### Expression analysis of endoplasmic reticulum chaperones, UPR transducers and autophagic related genes in Bz-treated larval stages

**Fig 2:**
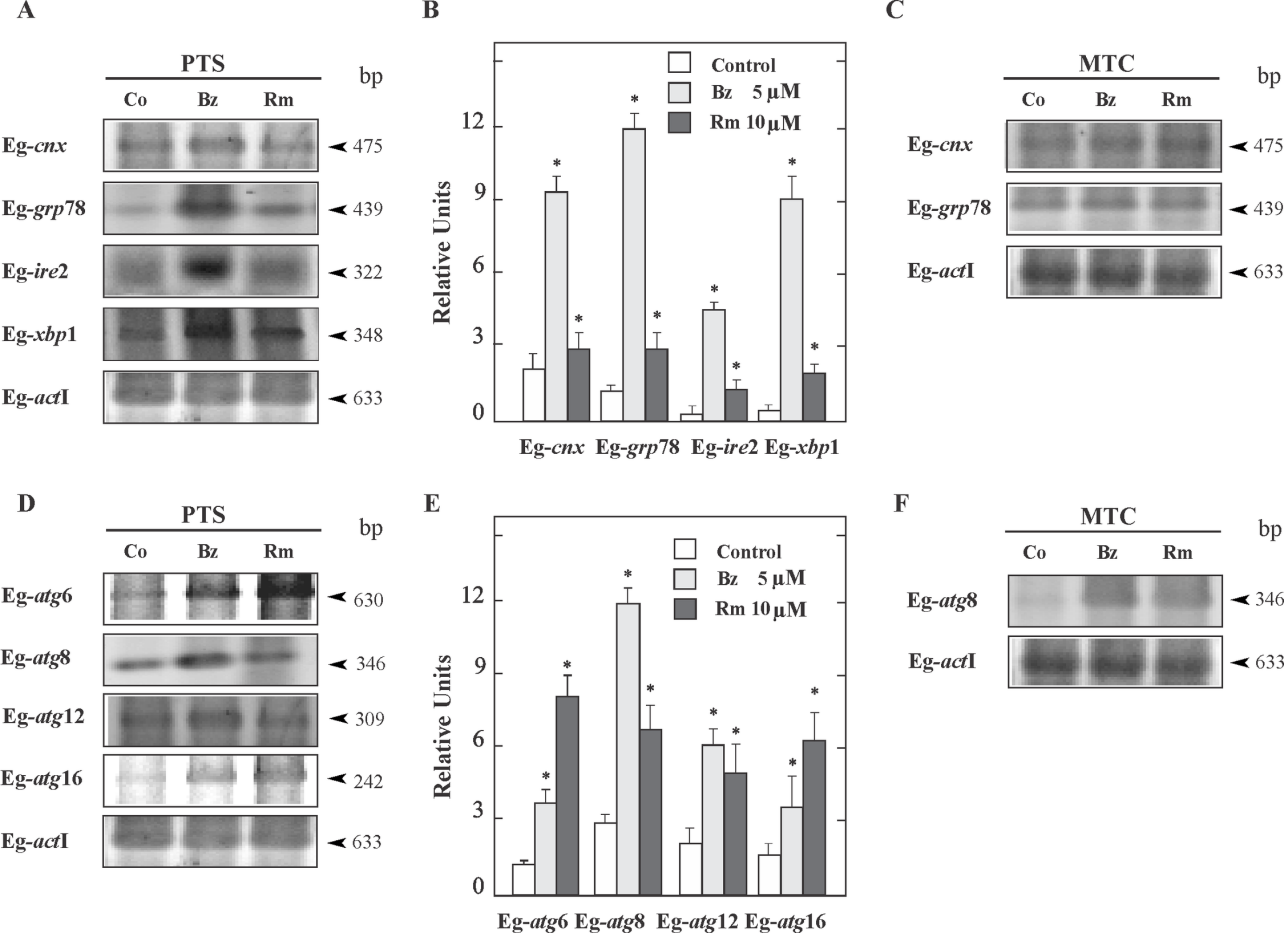
Expression of endoplasmic reticulum chaperones, UPR transducers and autophagic related genes in *E*. *granulosus* larval stages. (A) Reverse transcription (RT)–PCR analysis of Eg-*cnx*, Eg-*grp*78, Eg-*ire*2 and Eg-*xbp*1 from total RNA of protoscoleces (PTS) incubated for 48 h under control conditions (Co) or treated with 5 μM Bz or 10 μM Rm. Amplification of Eg-actin I (*act*I) was used as a loading control. Molecular sizes of amplicons are indicated with arrowheads. (B) Quantitative PCR was carried out under the same conditions as indicated in A. Values are means ± S.D. of three independent experiments. Asterisks indicate significant differences. (C) RT–PCR analysis of, Eg-*cnx* and Eg-*grp*78 from total RNA of metacestodes (MTC) incubated for 48 h under control conditions (Co) or treated with 5 μM Bz or 10 μM Rm. (D) RT–PCR analysis and (E) quantitative PCR of Eg-*atg* genes from total RNA of protoscoleces (PTS) carried out under the same conditions as indicated in A (F). RT–PCR analysis of Eg-*atg* genes from total RNA of metacestodes (MTC) carried out under the same conditions as indicated in C.

Perturbations of ER homeostasis by proteasome inhibition affect the protein folding and transcriptionally induce the expression of chaperons, which are considered indicators of ER stress. RT-PCR and RT-qPCR showed higher mRNA expression levels for Eg-*cnx* (three-fold) and Eg-*grp*78 (six-fold) in 5 μM Bz-treated protoscoleces during 48 h than in controls (Fig 2A and 2B). No changes were detected in the transcriptional expression of these genes in 5 μM Bz-treated metacestodes (Fig 2C). Thus, we demonstrated that up-regulation of gene expression of both ER molecular chaperones only occurs in protoscoleces. In addition, we analyzed the expression of Eg-IRE2/Eg-XBP-1 pathway, which is the highly conserved signaling branch of the UPR. RT-PCR and RT-qPCR showed that both Eg-*ire*2 and Eg-*xbp*1 were up-regulated nine and fifteen-fold in 5 μM Bz-treated protoscoleces, respectively in comparison to the controls (Fig 2A and 2B).

We also examined the expression level of autophagy-related genes from Bz-treated protoscoleces and metacestodes, which were standardized by comparison to *act* I expression. RT-PCR analysis indicated the increased expression of key Eg-*atg* genes in Bz-treated protoscoleces (Fig 2D). We found that the transcript levels for Eg-*atg*6, Eg-*atg*8, Eg-*atg*12 and Eg-*atg*16 increased two, three, two and one-fold with 5 μM Bz, respectively (Fig 2E) confirming the induction of autophagy. No changes were detected for Eg-*atg*1, Eg-*atg*2, Eg-*atg*3, Eg-*atg*4, Eg-*atg*5, Eg-*atg*7, Eg-*atg*9 and Eg-*atg*18 between control and Bz-treated protoscoleces and metacestodes (Fig 2D, S2 Fig and data not shown). All autophagy-related genes were expressed in metacestodes but only the transcript level for Eg-*atg*8 increased three-fold in Bz-treated metacestodes (Fig 2F and data not shown). Rapamycin was used as positive control in these experiments.

### Expression pattern of Eg-Atg8 and detection of autophagosomes in Bz-treated protoscoleces

**Fig 3:**
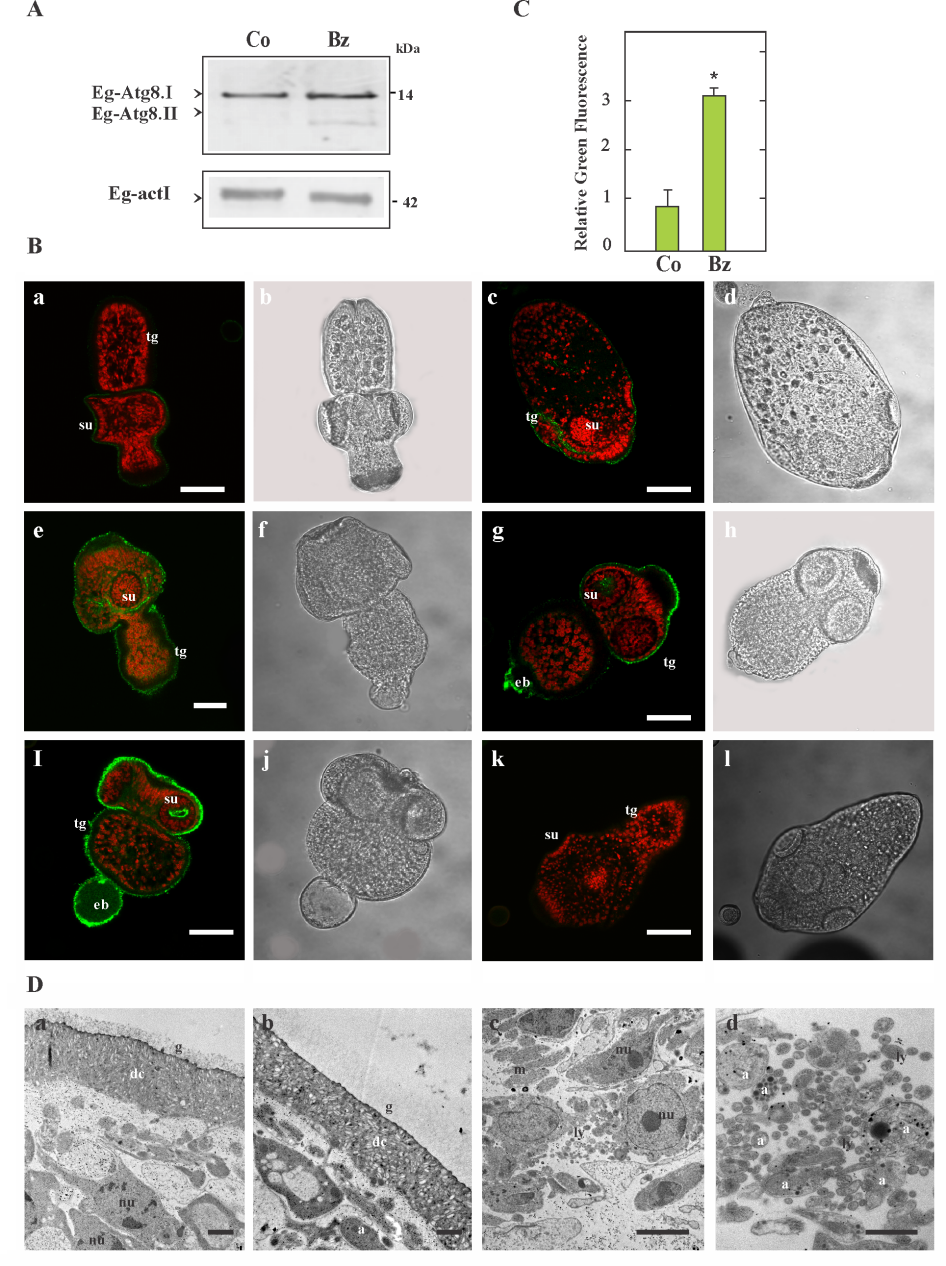
Immunolocalization of Eg-Atg8 and autophagosome detection from Bz-treated protoscoleces during 48 h. (A) Immunoblot of Eg-Atg8 revealed with an antibody against human LC3. Total protein extracts from control (Co) and 5 μM Bz-treated protoscoleces were loaded at 180 μg of total protein/lane. Both Eg-Atg8.I and PE-conjugated Eg-Atg8.I (Eg-Atg8.II) were detected. Anti-human-actin was used as loading control. Polypeptide sizes are shown. (B) Confocal images of *in toto* immunofluorescence assays performed with the same antibody anti-LC3 conjugated with Alexa 488 -green fluorescence- and counterstained with propidium iodide -red fluorescence-. Eg-Atg8 expression in control (a-d) and in Bz-treated protoscoleces (e-j). Negative control consisted of omission of primary antibody (k-l). Confocal images (a, c, e, g, i and k); transmission images (b, d, f, h, j and l); eb, excretory bladder; su, sucker; tg, tegument. Bars indicate 50 μm. (C) Normalized fluorescence intensity values of Eg-Atg8 expression in protoscoleces (control -Co- and Bz-treated -Bz-) are shown. Data represents three independent experiments with twenty parasites per experiment (n = 60). Scatter bars correspond to SD. *Statistically significant difference (P < 0.05) compared with controls. (D) Identification of autophagic structures in Bz-treated parasites by transmission electron microscopy (TEM). Soma from control protoscoleces exhibiting intact parasite tissue (a). In (b–d), the damage induced by Bz is shown with increased vesicularization of the glycocalix and the nuclei of tegumentary cytons exhibiting compacted cromatine (b). Note the high number of lysosomes (ly) in the subtegument with its characteristic electron-dense oval structure (c). In (d) a high-magnification image allows detecting autophagosomes (a, double membrane vesicles with engulfed cytosolic content) and autolysosomes (single membrane vesicles). g, glycocalyx; dc, distal cytoplasm; nu, nucleus; m, mitochondria. Bars indicate 1μm (a-c) and 0.5μm (d).

Given that Atg8 controls the size of the autophagosome, its lipidation is widely used to monitor autophagy induction [35]. Therefore, using a rabbit polyclonal antibody, the Eg-Atg8.I (soluble) and Eg-Atg8.II (Eg-Atg8.I conjugated to phosphatidylethanolamine–PE-, lipidated and faster migrating protein) could be detected by immunoassays in control and Bz-treated protoscoleces. Our results showed that both bands were more intense in the drug-treated sample (Fig 3A). The bands were not observed when the strips were incubated with the secondary antibody alone (data not shown).

*In toto* immunofluorescence labeling showed that anti-Atg8 reactivity was localized in the cytoplasm of tegumental cells, suckers and excretory bladder of protoscoleces (Fig 3Ba-j). Protoscoleces treated with 5 μM Bz during 48 h showed a two-fold increase in the fluorescence intensity compared to the control (Fig 3Be-j and 3C). No signal was detected in the control sections that were only incubated with secondary antibody under the same conditions (Fig 3Bk). Non-specific fluorescent signal was detected in the rostellar hooks as previously described (Fig 3Bc-l, [28]).

These results were confirmed at the ultrastructural level by TEM. The tegument ultrastructure and its associated glycocalyx in control protoscoleces appeared unaltered (Fig 3Da). At 2 days post incubation, Bz-treated protoscoleces revealed the internal tissue altered with the presence of numerous autophagic structures. Note the changes in the increased vesicularization of the glycocalix and altered mitochondria in Bz-treated protoscoleces (Fig 3Db). Major effects of Bz include the appearance of several lysosomes (Fig 3Dc-d), autophagosomes (limited by a double-membrane) and autophagolysosomes containing stacks of electron-dense (constrained by a single membrane, Fig 3D-d), both containing cytoplasmic content.

### Bortezomib and rapamycin synergistically induced cytotoxicity in *Echinococcus* larval stages

**Fig 4:**
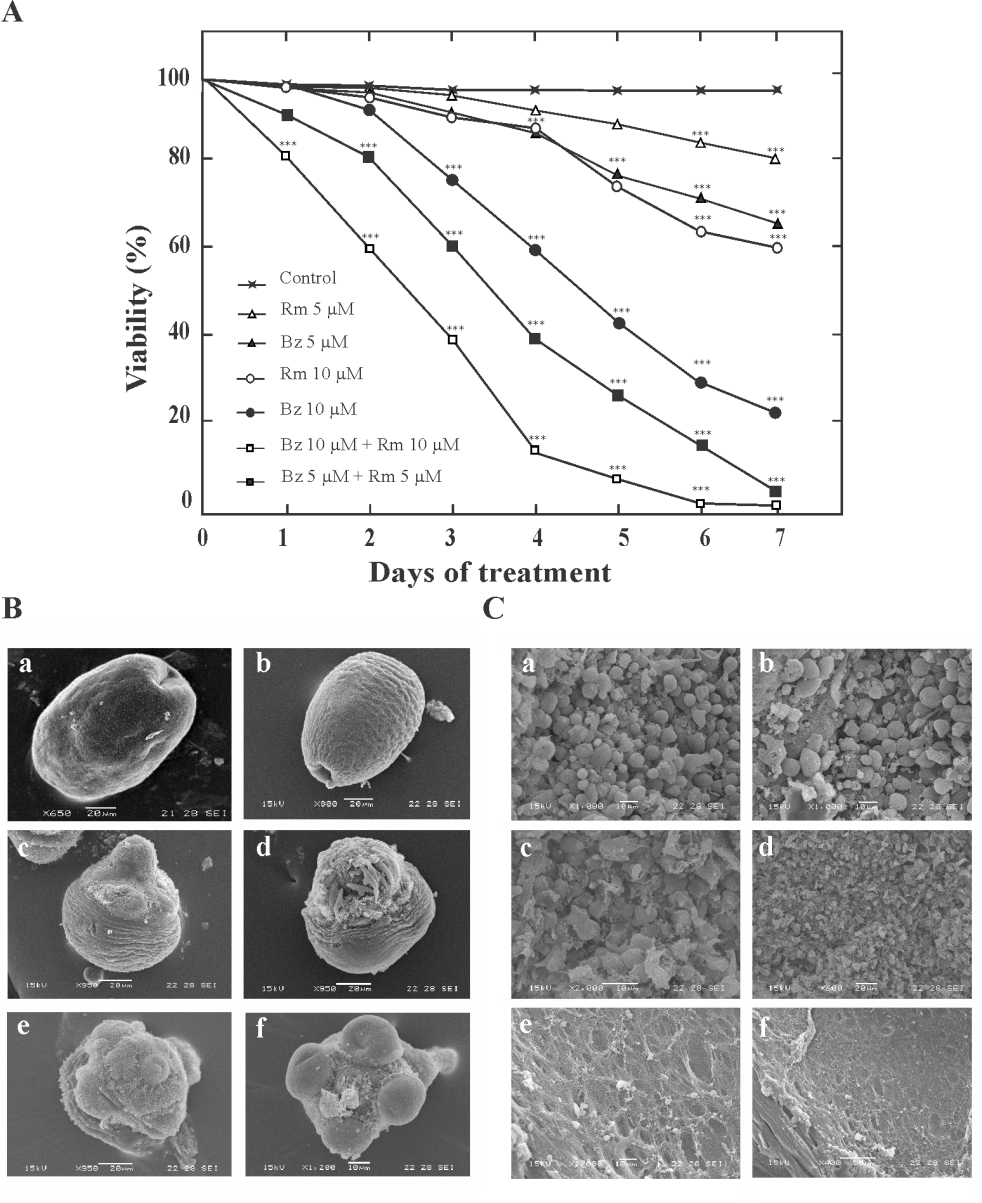
Effect of combinatorial treatment of bortezomib (Bz) and rapamycin (Rm) on *Echinococcus* cell viability. (A) Effect of Rm and Bz on *E*. *granulosus* protoscolex viability at 10 μM for individual drugs and 5 μM or 10 μM of each drug for a combined protocol over 7 days (the drugs were replenished after 3 days). Protoscoleces incubated in culture medium containing 1:1.000 DMSO were used as controls. Each point represents the mean percentage of vital protoscoleces from three different experiments. Asterisks indicate a statistically significant difference (P < 0.05) from the corresponding control. (B) Ultrastructural changes of protoscoleces detected by SEM after pharmacological treatment for 48 h. Control (a-b), 5 μM Bz (c-d), 5 μM Bz + 5 μM Rm (e-f). Treated protoscoleces presented contraction of soma region, alteration of tegument, and scolex region showing loss of hooks and shedding of microtriches. Bars indicate 10μm in (f) and 20 μm in (a-e). (C) Ultrastructural damage of metacestodes detected by SEM after pharmacological treatment for 48 h. Control (a-b), 0.5 μM Bz (c-d), 0.5 μM Bz + 5 μM Rm (e-f). Ultrastructural alterations appeared earlier than germinal layer detachment in metacestodes treated with 5 μM Bz. Bars indicate: 10μm in (a-c, e), 20 μm in (d) and 50 μm in (f).

To determine the impact of autophagy induction on *Echinococcus* cell viability, combinatorial treatment with Bz and Rm was investigated. Synergistic effect on protoscolex viability was found using a combination of Bz plus Rm (Fig 4A). All protoscoleces treated with 5 μM Bz plus 5 μM Rm were dead after 7 days in culture, whereas 5 μM Bz or 5 μM Rm only reduces 35% and 20% the protoscolex viability after 7 days (Fig 4A). Regarding with the ultrastructural study by SEM, after 48 h of treatment, the ultrastructure of protoscoleces and metacestodes incubated with 5 μM and 0.5 μM Bz (Fig 4Bc-d and 4Cc-d), respectively exhibited significant differences compared with control samples (Fig 4Ba-b and 4Ca-b). Bz-treated protoscoleces showed the tegument completely altered, shedding of microtriches, loss of hooks, and contraction of soma region. Bz-treated metacestodes presented loss of cells from the germinal layer of cysts. In combinatorial treatments, protoscoleces showed severe tegumental injuries and metacestodes revealed only cell debris in their germinal layers, with 5 μM Rm plus 5 μM or 0.5 μM Bz respectively, after 48 h of treatment (Fig 4Be-f and 4Ce-f). Thus, the dual drug sensitivity suggests that the combination therapy promoted autophagy and synergistic antiechinococcal effects.

## 4. Discussion

The *Echinococcus* genome decoding [19–20] has provided powerful tools to find new drug targets with potential use in chemo-treatment. In accordance with the findings reported by Tsai et al. [19], Stadelmann et al. [18] have described *in vitro* and *in vivo* anthelmintic activity of Bz in metacestodes of *E*. *multilocularis*. In contrast to *E*. *multilocularis* where the protoscolex development has been rarely described in humans [36], secondary echinococcosis can be induced by dedifferentiation of *E granulosus* protoscoleces [37]. Thus, this larval form has also been included in our *in vitro* study, where it was reported a deleterious effect of Bz in both protoescoleces and metacestodes from *E*. *granulosus*. Additionally, we demonstrated that Bz induces the transcriptional expression of chaperones and promotes the activation of the UPR pathway and autophagy as a mechanism to counteract the stress generated in the parasite.

In the first place, our results evidenced the potent pharmacological and dose-dependent effect of Bz on *E*. *granulosus* protoscoleces and metacestodes from a concentration of 5 and 0.1 μM, respectively (Fig 1). Bortezomib treatment of protoscoleces (~15 μM) and metacestodes (1 μM) resulted in the killing of 50% of parasites compared with the controls after 96 h of exposure to the drug. This viability decrease occurs at earlier time points in comparison, with classical antiechinococcosic drugs [38]. Similarly, previous studies on *E*. *multilocularis* metacetodes showed a high viability reduction at low Bz concentrations (0.6 μM for 5 days) due to the effective proteasome inhibition and the accumulation of poly-ubiquitinated proteins [18]. As it has been previously reported, the parasite proteasomes could be possible targets because they are involved in the regulation of cell differentiation, proliferation, encystation, larval invasion and interaction with hosts [39–41].

Proteasome inhibition triggers the UPR induction [4]. The UPR transducers have increased in number along the eukaryote evolution, starting with only IRE1/XBP-1 signaling in *Saccharomyces cerevisiae*. In *Caenorhabditis elegans* and *D*. *melanogaster* the three branches of the UPR are coded in their genome, but only two are functional (IRE1/XBP-1 and PEK1) [42], while in mammals IRE1, PERK and ATF6 pathways regulate homeostasis in the ER [42–43]. Particularly intracellular parasites, such as *Leishmania*, *Toxoplasma* and *Trypanosoma* in absence of ER sensors IRE1/XBP-1 and ATF6, equally undergo UPR through recognition of misfolded protein by PERK [44]. According to Mori et al [42], *Echinococcus* sp. might have all these functional proteins. Bioinformatic and transcriptome analysis from *Echinococcus* genome [20] allowed us the identification of an IRE2 (EgrG_000182000) and a XBP-1 (EgrG_000843400.1) ortholog. We have also identified the putative ortholog for ATF6 (EgrG_000116500.1). Nevertheless the branch PEK1/PERK-ATF4 was not found in the genome (S1 Fig). In homeostatic conditions *Echinococcus* cells, maintain basal levels of IRE/XBP-1 [20]. Recently it has been described a critical role of XBP1 in ER-Golgi biogenesis and the development of highly secretory exocrine cells [45–46]. The level of IRE2/XBP-1 mRNA (Fig 2A and 2B) could explain the high rate in the exocrine activity of the parasite [47]. Also, in Fig 2A and 2B we showed by RT-PCR and RT-qPCR the expression of genes of UPR signaling IRE2 and XBP-1 in protoscoleces of *E*. *granulosus* and we have observed an up-regulation after Bz treatment, proving that the deleterious effects of Bz on the parasite come from an exacerbated activation of the UPR which drives to parasite death. The presence and expression of this stress sensor in *E*. *granulosus* as an UPR signal conserved from yeast to mammals [48], provides a starting point but further characterization of this process in cestodes is needed.

In ER-stress conditions, cells promote the transcriptional activation and over-expression of chaperones to mitigate the increase in the misfolding protein rate [49–52]. In *Echinococcus* sp., the chaperone, Eg-Grp78, has been described as an important parasitic antigen [23] and it was found in five different spliced variants in adult and protoscolex forms [53]. In this work it has been demonstrated that Eg-*grp78* and Eg-*calnexin* genes showed high basal expression levels in both larval forms during normal growth conditions, and only showed a notable gene up-regulation in protoscoleces in response to Bz treatment.(Fig 2A and 2C) evidencing the onset of ER stress and UPR activation. Surprisingly, in metacestode cells, where protein synthesis rate exceeds that of the protoscoleces, the increasing expression of these molecular chaperones in response to Bz was not evident. Therefore, in this larval form the low expression of chaperones (Fig 2C) and UPR transducers [20] could explain the high susceptibility to Bz at low concentrations (Fig 1B). These results correspond with the transcriptome data, where has been reported a very low expression of Eg-*ire* in cyst larval form (identified as EgR_04887 in [20]). In this line of evidence, it has been demonstrated that, *ire-*1 deficiencies reduce the apoptosis in germline cells from free life worms [54], and that *ire*1α −/− mouse embryonic fibroblasts exhibit a greater survival rate than wild type cells under ER stress, supporting the concept that IRE1 in stress conditions is an apoptotic trigger protein [55]. Therefore it will be interesting to investigate the IRE functions in *Echinococcus* metacestodes. Additionally, Grp78, also binds to ER-associated caspase 7 and caspase 12 and suppresses their activation, thus result to have not only protein folding capacity, but also act as a potent pro-survival protein in cells in stress conditions [52]. For this reason, a low expression of Eg-*ire* and a high Eg-*grp*78 expression could account for a reduced capacity to elicit apoptosis in the germinal cells of the cestode. On the other hand, high basal expression of chaperones in protoscoleces could be related to the regulation of Ca^2+^ homeostasis, an important process in this parasite [27, 30]. Also, calnexin (shown in Fig 2) and calreticulin can regulate the quality control of glycoproteins in the ER, which are crucial to generate the laminar layer in *E*. *granulosus* [2].

Given that different reports have observed a crosstalk between the proteasome-mediated protein processing and the photolytic process of autophagy [56–57], other aim of this work was to study the autophagy induction in Bz-treated parasites. So, following the proteasome inhibition, we demonstrated a transcriptional induction of autophagic genes for both larval forms (Fig 2D and 2F) in comparison with the control counterpart. Parasite death has been preceded by a raise in the levels of Eg-Atg8 (Fig 3A and 3C), which has been used as an autophagy induction marker [58]. The process of autophagosome formation depends on several autophagic proteins [28, 59], but by post-translational modification of soluble Atg8-I, Atg8-II localizes exclusively in the autophagosomal membranes allowing us to detect by confocal microcopy a dot pattern of Eg-Atg8 in Bz-treated protoscoleces (Fig 3Be-j) and an increased number of autophagosomes revealed by the ultrastructural analysis (Fig 3Dc-d). Bortezomib significantly increased the conversion of Atg8-I to Atg8-II in *E*. *granulosus* as has been described in different cell systems [60–62]. Moreover, in order to clear the ER from the accumulation of terminally misfolded protein aggregates due to the proteasome inhibition, the UPR may up-regulate the autophagy machinery [29, 63–64]. However, when persistent ER stress could not be mitigated, this process can switch the cytoprotective functions of UPR and autophagy into cell death promoting mechanisms. Autophagy directly correlates with Bz-induced ER stress in several ways, including the up-regulation of Atg6/beclin [65], the activation of c-Jun NH_2_-terminal kinase (JNK) through IRE/XBP-1 arm of the UPR (resulting in Bcl-2 phosphorylation and impairment of Bcl2/beclin1 interaction [66–67]), the Grp78 over-expression [68] and/or the dephosphorylation of mTOR [69]. Our results are concordant with these data, because we demonstrated the transcriptional up-regulation of the autophagic gene Eg-*atg*6, the chaperone Eg-*grp78* and the IRE/XBP-1 pathway in Bz-treated protoscoleces (Fig 2A, 2B and 2D–2E).

Finally, given the controversial data about pro-survival and cytotoxicity in the combination of Bz with an autophagy inducer, such as rapamycin [67, 70], we analyzed the joint action of both drugs in the larval stage of *Echinococcus*. We found that the combination of Bz with rapamycin enhanced the rate of cell death in protoscoleces and metacestodes (Fig 4). Not only do these findings further illustrate the remarkable regulatory power of proteasome activity and the importance in the UPR pathway, but also could reveal an unexpected high capacity of autophagy induction in *Echinococcus* cells.

## 5. Conclusions

This study showed the lethal effect of Bz against the larval stage of *E*. *granulosus*. Also, it was determined a mRNA increase of ER-chaperones and UPR transducers as a consequence of the ER-stress generated by Bz in protoscoleces. While in metacestodes, these pathways were not upregulated preventing the homeostasis restore and enhancing their vulnerability. Furthermore, we determined autophagic-related gene expression, Eg-Atg8 membrane association and observed autophagosome formation as a mechanism that finally drives to parasite death. Thus, knowledge of the mechanisms associated with stress and its regulatory pathways with pharmacological compounds will be of relevance in the design in the near future of new therapeutic strategies for hydatidosis.

## Supporting information

S1 Fig Structural organization of the *Echinococcus* molecular chaperones and unfolded protein response (UPR) proteins based on the functional domains of the human orthologs. Protein identity and accession numbers or genome protein denomination are indicated in the left column. Protein length is indicated from N-terminal (1) to C-terminal end and indicated as “L” or “C” the luminal or exoplasmic or outer (cytosolic) faces of the ER, respectively. A comparison of identity percentages with different queries in the right column. *E*. *granulosus* (Eg), *H*. *sapiens* (Hs) and *D*. *melanogaster* (Dm). **Eg-Grp78/BiP** (Glucose-regulated protein 78) chaperone presents at N-terminal an ATPase domain (ATPaseD, light green) which is necessary for its anti-apoptotic function, a substrate-binding domain (SBD, red) related to protein-refolding activity and the KDEL C-terminal motif an ER retention/retrieval sequence to keep the chaperones in the ER. [22]. **Eg-Calnexin (Eg-Cnx)**, consists of a globular N-terminal domain (GND, residues 1–200, blue box) which contains a Ca^2+^ binding and the glucose-binding site or lectin domain required for the N-glycosylation reactions in the ER [71], a proline-rich tandem sequence named the P domain (PD, residues 250–440, yellow box), and at C-terminal, a transmembrane domain (TMD, black bar) due to it is an integral membrane protein of the ER. **Eg-Calreticulin (Eg-Clr)**, is a luminal protein that lacks membrane binding domains, which is able to travel freely within the ER lumen. Also, it conserves the characteristic N-terminal lectin domain (blue bar) and the extended arm P-domain (yellow bar), which may form a functional “protein-folding module” in association with the same calnexin domains. The C-terminal region of Eg-Crt is highly acidic and it may bind Ca^2+^ with high capacity involving its storage in the lumen of the ER [72]. **Eg-IRE** (Inositol-requiring protein,the most essential and conserved ER stress sensor of the UPR in eukaryotes) [5]. It has the characteristic N-terminal luminal dimerization domain (NLD, indicated in red, that comprises S^24^-V^390^ residues in Hs-IRE1α), a BiP-binding site (BBS, blue box, conserved region D^475–526^ in Hs-IRE1α) for interacting with Grp78/BiP within the ER. After Grp78/BiP release, and its binding to accumulated unfolded proteins, IRE undergoes conformational changes promoting its cytosolic kinase activity through the serine:threonine kinase domain(STK, orange bar) which has a K^599^conserved residue (asterisk), essential for this enzyme activity [73], and in addition, activating its C-terminal endoribonuclease activity (RNase domain indicated with a grey bar and with the characteristic conserved motif -Yx_6_DLLx_3_RNx_2_HHx_21_Yx_4_FLxL, [74]). This last enzyme activity is required for the cleavage of the Xbp1 mRNA. **Eg-XBP1** (X-Box protein 1) is a transcription factor which activates the UPR in metazoans (pfam07716, [5]). This predicted unspliced protein, is a prototypical basic leucine zipper transcription factor (bZIP region, indicated with a red bar) with a DNA-binding motif (14 conserved residues, ^37^EEKVLRRKLLNREAA^51^) plus a dimerization domain with a hydrophobic region (HR, green box) important for proper localization of Xbp1 mRNA at the ER membrane. It is a highly conserved protein in *Echinococcus* sp. but lacks of translational pausing domain present in orthologous proteins (TP, white box in Hs-XBP-1) with regulatory functions [48]. **Eg-ATF6** (Activating Transcription Factor 6), is other UPR transcription factor that, has a basic leucine zipper domain (bZIP, light blue box), with the characteristic motif (-R/Kx_3_Nx_2_AA/Qx_2_F/YR-). It is anchored by a transmembrane domain (TMD indicated as a black box) at the ER in unstressed cells. In cells undergoing ER stress, ATF6 is processed releasing its cytosolic domain fragment, which controls the upregulation of genes encoding XBP1 and ER-associated degradation (ERAD) components.
(TIF)

S2 Fig Expression of autophagic related genes with no changes in *E*. *granulosus* protoscoleces. Reverse transcription (RT)–PCR analysis of Eg-*atg* genes from total RNA of protoscoleces (PTS) incubated for 48 h under control conditions (Co) or treated with 5 μM Bz or 10 μM Rm. Amplification of Eg-actin I (*act*I) was used as a loading control. Molecular sizes of amplicons are indicated with arrowheads.
(TIF)
