# Supplementary figures and images for: Bortezomib initiates endoplasmic reticulum stress, elicits autophagy and death in Echinococcus granulosus larval stage
Source: PLoS One. 2017 Aug 17;12(8):e0181528. doi: 10.1371/journal.pone.0181528 (PMC5560652; doi:10.1371/journal.pone.0181528)

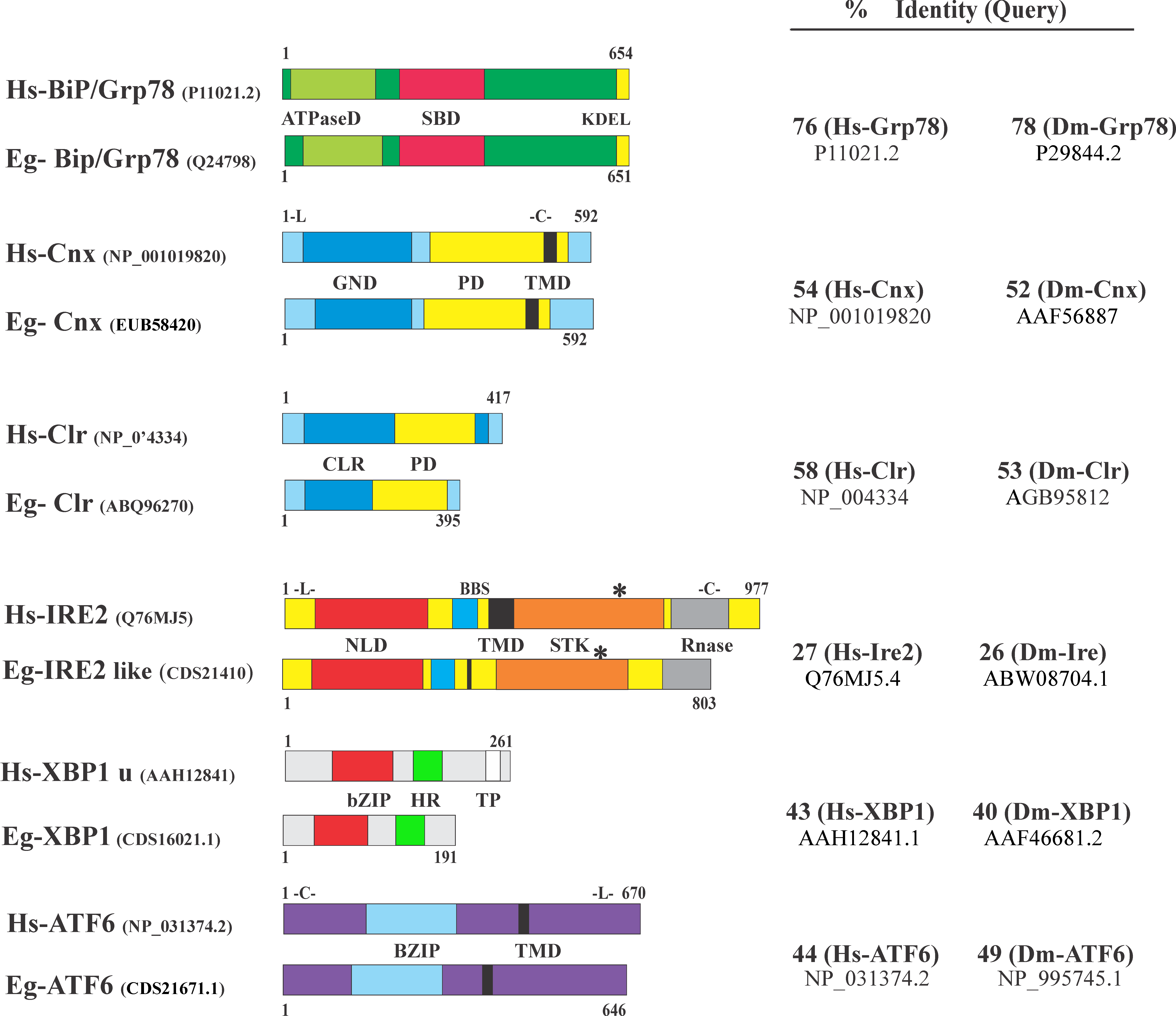

Supplement: S1 Fig — Protein identity and accession numbers or genome protein denomination are indicated in the left column. Protein length is indicated from N-terminal (1) to C-terminal end and indicated as “L” or “C” the luminal or exoplasmic or outer (cytosolic) faces of the ER, respectively. A comparison of identity percentages with different queries in the right column. E. granulosus (Eg), H. sapiens (Hs) and D. melanogaster (Dm). Eg-Grp78/BiP (Glucose-regulated protein 78) chaperone presents at N-terminal an ATPase domain (ATPaseD, light green) which is necessary for its anti-apoptotic function, a substrate-binding domain (SBD, red) related to protein-refolding activity and the KDEL C-terminal motif an ER retention/retrieval sequence to keep the chaperones in the ER. [22]. Eg-Calnexin (Eg-Cnx), consists of a globular N-terminal domain (GND, residues 1–200, blue box) which contains a Ca2+ binding and the glucose-binding site or lectin domain required for the N-glycosylation reactions in the ER [71], a proline-rich tandem sequence named the P domain (PD, residues 250–440, yellow box), and at C-terminal, a transmembrane domain (TMD, black bar) due to it is an integral membrane protein of the ER. Eg-Calreticulin (Eg-Clr), is a luminal protein that lacks membrane binding domains, which is able to travel freely within the ER lumen. Also, it conserves the characteristic N-terminal lectin domain (blue bar) and the extended arm P-domain (yellow bar), which may form a functional “protein-folding module” in association with the same calnexin domains. The C-terminal region of Eg-Crt is highly acidic and it may bind Ca2+ with high capacity involving its storage in the lumen of the ER [72]. Eg-IRE (Inositol-requiring protein,the most essential and conserved ER stress sensor of the UPR in eukaryotes) [5]. It has the characteristic N-terminal luminal dimerization domain (NLD, indicated in red, that comprises S24-V390 residues in Hs-IRE1α), a BiP-binding site (BBS, blue box, conserved region D475– [file pone.0181528.s001.tif]

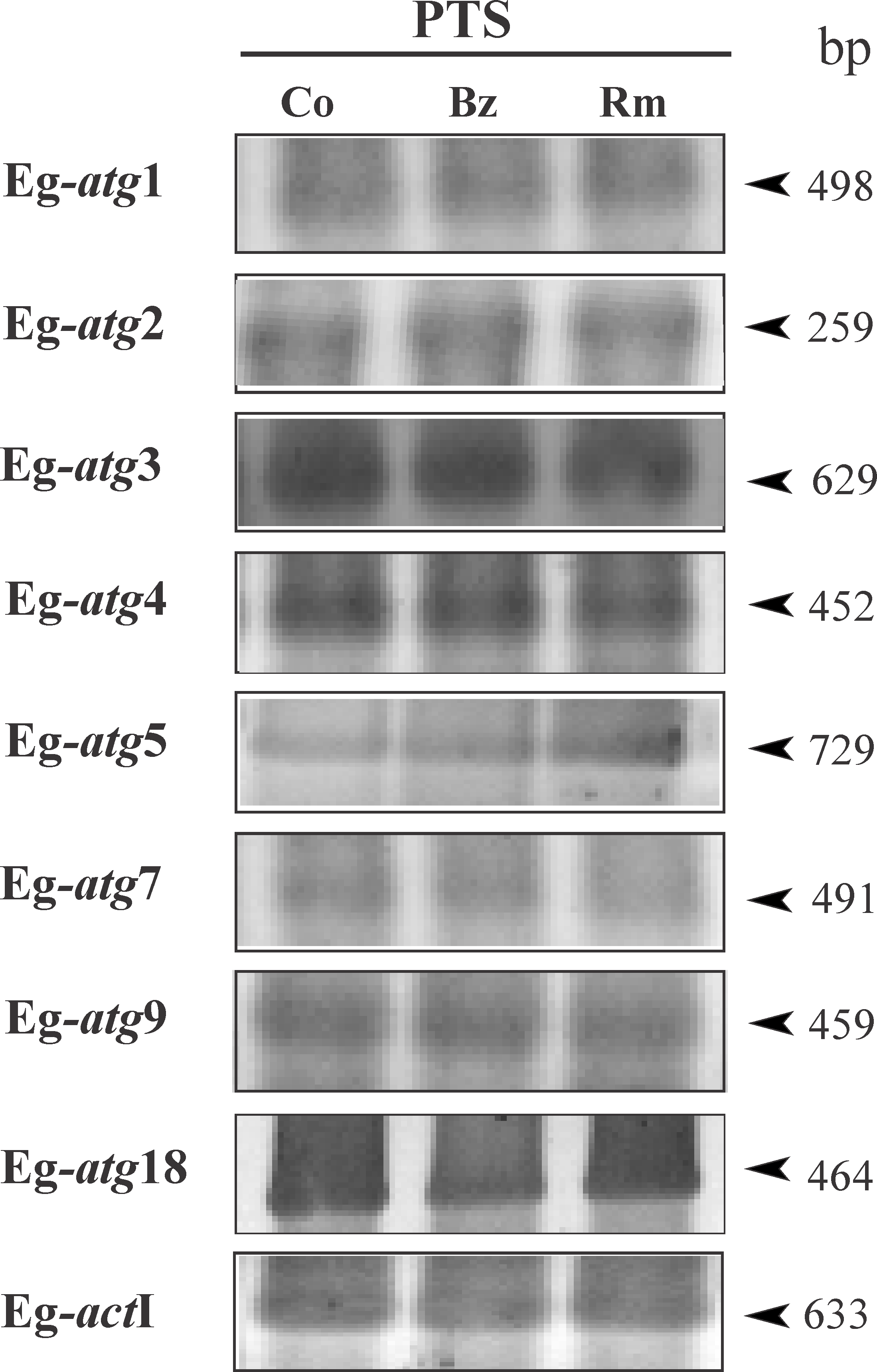

Supplement: S2 Fig — Reverse transcription (RT)–PCR analysis of Eg-atg genes from total RNA of protoscoleces (PTS) incubated for 48 h under control conditions (Co) or treated with 5 μM Bz or 10 μM Rm. Amplification of Eg-actin I (actI) was used as a loading control. Molecular sizes of amplicons are indicated with arrowheads. (TIF) [file pone.0181528.s002.tif]
